# The B.M.A. and the Law of the Land

**Published:** 1918-10-26

**Authors:** 


### THE B.M.A. AND THE LAW OF THE LAND.

The judgment delivered in the King's Bench Division by Mr. Justice McCardie, on the 15th inst., raises a very acute position for the whole of the medical profession. The immediate penalties, in the shape of damages and costs, fall of course on the defendants in the action, namely, the British Medical Association and certain doctors practising at Coventry. But the situation created by the judgment is one to which every member of the Association must needs give heed, and even non-members have their concern in the debate. What has to be decided is whether the Association shall be supported or repudiated, and whichever way the vote is cast it is likely to have serious consequences.

The essential facts which led to the legal struggle can be briefly stated. For many years there has existed at Coventry a Provident Dispensary, and the conduct of this institution has long excited professional discontent. So far back as 1907 the position became an acute one, and the medical staff, their remonstrances not being accepted, resigned office. In this step they were supported by the local division of the British Medical Association, and the dispensary was condemned as an institution which no medical practitioner ought to be willing to serve. A certain number of practitioners, however, took a different view and, coming to practise in the neighbourhood, they accepted appointments on the dispensary staff and carried on the work. In the nature of things this action incurred the hostility of the medical opponents of the dispensary, and the new officials were formally declared to have acted "contrary to the honour and interests of the profession." To this verdict the central authorities of the British Medical Association gave their endorsement and support, and under their authority the penalties prescribed by the ethical rules of the Association were brought into active operation. The procedure included the circulation among practitioners of the conclusion that the doctors in question were acting contrary to "the honour and interests of the profession," and a decree that for any practitioner to hold any professional relations with them involved the risk of a kindred anathema. The new men, in short, were to be isolated, and to be ostracised from all professional intercourse. It was the application of these decisions that led to an action by the condemned practitioners, and to a claim for damages for "conspiracy to injure them in their profession and to libel and slander them," and the dispute has now been decided in their favour.

The most important consequence of the decision, at least so it seems to us, is the intimation that the procedure justified and even advised in the case of ethical disputes by the rules of the British Medical Association includes steps which are now authoritatively declared to be contrary to the law of the land. The Coventry Dispensary may be all that the former officers of the institution say it to be, and the men who took service on its staff may be quite without professional justification for their action. But even when these propositions are admitted it still remains true that the methods adopted to punish the offenders have been proved to be illegal. Now for these measures the Association is responsible, and they are indeed prescribed and encouraged by the rules of the Association. These rules stand, therefore, at the present moment as a defiance of the law of the land, and neither the Association nor any of its members will venture to put them into operation. From such a situation there can surely be only one issue. The rules which in practice have led the Association to humiliation and condemnation in the Law Courts must be cancelled and withdrawn, and it is difficult to see how their promoters and defenders can be allowed to retain their present positions of authority and responsibility. The whole proceeding has been a deliberate one, and it has resulted in what can only be described as a serious slur on the name and reputation of the Association, and a definite prejudice to its influence and usefulness. Certainly the Association must at once take steps to recognise the profound error into which it had been led, and must set itself right both with the public and with the profession. Confession, retraction, and amendment are called for, and we trust they will be forthcoming. If not, the whole future of the organisation would seem to be in peril, for it is impossible to believe that members will continue their allegiance to a body which encourages by its rules a line of procedure judicially condemned as illegal and improper.

How the present position has been created would involve a prolonged inquiry into recent history. It is, however, safe to say that many members of the Association are probably quite unaware of the existence on the official chart of the methods which have now led to such dire disaster. These have been developed and organised by an energetic section anxious to secure the power to inflict penalties on those who pretend to defy the Association's decrees. Warning voices were uttered from the outset, but these were disregarded. In the debates many members took no interest, or no sustained interest, with the result that the aggressive and penalising advocates gained their way. Now interest is compelled, for a responsibility which has hitherto been a corporate one takes on an individual application, and the private member must face the issue. Officials and rules are in the last analysis his creation, and as he has the power to shape them he cannot escape the consequences of his ability. There are still wider issues raised by the recent judgment, and these we shall hope to discuss at a later date. Our immediate concern is with the narrow but highly important point that certain rules of conduct standing as approved by the British Medical Association have been condemned as contrary to the law of the land.

